# The complete plastid genome of *Terminalia myriocarpa* Vaniot Huerck et Muell.-Arg (Combretaceae), a tropical rainforest indicator species in Southern China

**DOI:** 10.1080/23802359.2022.2073848

**Published:** 2022-05-10

**Authors:** Xiao Yu, Zhen-Ning Zhao, Huai-Lei Ping, Li-Lan Deng

**Affiliations:** a School of Landscape Architecture and Horticulture Sciences, Southwest Forestry University, Kunming, China; b School of Forestry, Southwest Forestry University, Kunming, China

**Keywords:** *Terminalia myriocarpa*, plastid genome, phylogenetic analysis

## Abstract

*Terminalia myriocarpa* Vaniot Huerck et Muell.-Arg is a tropical rainforest indicator species in Southern China. The chloroplast genome of *T. myriocarpa* was analyzed by high-throughput sequence technology, and its genetic relationship to related species was discussed. The chloroplast genome is 159,854 bp in length, with a total GC content of 37%. It has a typical chloroplast tetrad structure, including 88,015 bp of large single copy (LSC), 18,814 bp of small single copy (SSC), and 26,319 bp of inverted repeats (IR). A total of 130 genes were annotated, including 85 protein-coding genes, 8 rRNA genes, and 37 tRNA genes. Phylogenetic analysis indicated *T. myriocarpa* was closely related to *Terminalia phillyreifolia.*

*Terminalia myriocarpa* Vaniot Huerck et Muell.-Arg(1870) is an evergreen or semi-evergreen tree belonging to the genus Terminalia of Combretaceae family. It is mainly distributed in the Southern Guangxi Zhuang Autonomous Region, South Central in Yunnan Province, and Southern Tibet Autonomous Region of China (Wu 1984). This species is not only one of the common upper tree species in the production area but also an important part of the wild plant resources in the northern subtropics. In China, *T. myriocarpa* is classified as a second-grade protected wild plant in the Chinese Plant Red Book. Meanwhile, it is classified as endangered according to the IUCN (International Union for Conservation of Nature) Red List of threatened species (Li 2004). Although *T. myriocapra* is widely distributed in the tropical or southern subtropics of China, the number of existing wild individuals has a very low quantity. The study for *T. myriocapra* has been focused on the seedling technique (Lu et al. 2010; Yu GX et al. 2017; Yu et al. 2022) and endangered protection (Yu X et al. 2017; Yu et al. 2019, 2020), there was no record of complete plastid genome sequence to date. In this study, we characterized a complete plastid genome of *T. myriocapra* and confirmed the phylogenetic relationship of the genus, to provide genetic information for further research on phytogeography, genetic diversity, and evolution.

Fresh leaves of *T. myriocarpa* were collected from Ailao Mountain National Nature Reserve, Zhenyuan County, Yunnan Province, China (101°25′38.58″E, 23°56′8.00″N,1792m). The collection of plant materials was in accordance with local regulations and obtained the permission of local authorities. A voucher specimen (SWFU20210721MFY) was deposited in the Herbarium of Southwest Forestry University, China (http://bbg.swfu.edu.cn/, Yu Xiao, email: yuxiao0215@gmail.com). Total genomic DNA was extracted from silica gel dried leaf tissues using a modified CTAB method (Doyle and Doyle 1987). A total of 3 G raw data from Illumina Hiseq Platform (Illumina, San Diego, CA) were sequenced. GetOrganelle program was used to assemble the original data, and the parameters were: wordize = 102; base coverage = 171.44; *k* = 75, 85, 95, 105, 115, 127 (Jin et al. 2020). Annotation using Geneious Prime (Kearse et al. 2012) with reference to the complete plastid genome sequence of *Terminalia catappa* (NC_053323). The complete plastid genome of *T. myriocarpa* was submitted to GenBank with accession number OM202511.

The complete plastome of *T. myriocarpa* is 159,467 bp in length with a typical double-stranded circular tetrad structure, containing a large single-copy (LSC) region of 88,015 bp, a small single-copy (SSC) region of 18,814 bp, and a pair of inverted repeat (IR) regions of 26,319 bp each. The overall GC content of the whole genome is 37%. GC content in the IR region (43.02%) was higher than that in the LSC region (34.80%) and SSC region (30.65%). In total, 130 genes were annotated in the plastome, including 85 protein-coding genes (PCGs), 8 ribosomal RNA genes (rRNAs), and 37 transfer RNA genes (tRNAs). A total of 101 SSRs were discovered by the online software MISA-web (Beier et al. 2017). Among them, the numbers of mono-, di-, tri-, tetra-, and pentanucleotides SSRs are 86, 5, 5, 5, and 0, respectively.

The phylogenetic tree was reconstructed based on chloroplast genome sequences of *T. myriocarpa* and 11 species of Combretaceae. *Malus pumila* and *Rosa roxburghii* were used as outgroups. MAFFT v.7 program: scoring matrix = 200, PAM *k* = 2, gap open penalty = 1.53, offset value = 0.123 (Katoh and Standley 2013) was used to make a multiple alignments of the chloroplast genome sequences of these 15 plants. Then the alignment results were checked in Geneious 11.0.3 software, and the *.net format file was output. ML tree constructed with RAxML ver.8.0.0 software: *m* = GTR + GAMMA, Bootstrap = 1,000 (Stamatakis 2014). The maximum-likelihood phylogenetic tree was visualized using FigTree 1.4.3 software (http://tree.bio.ed.ac.uk/software/figtree/). The phylogenetic analysis revealed that all species of Combretaceae formed one clade. The phylogenetic tree showed that *Terminalia phillyreifolia* was sister to *T. myriocarpa* with strongly supported under current sampling (Figure 1). The complete chloroplast genome of *T. myriocarpa* will be used for population genomics research, phylogenetic analysis, and genetic engineering research, which will contribute to the better development and utilization of this species.

**Figure 1.**
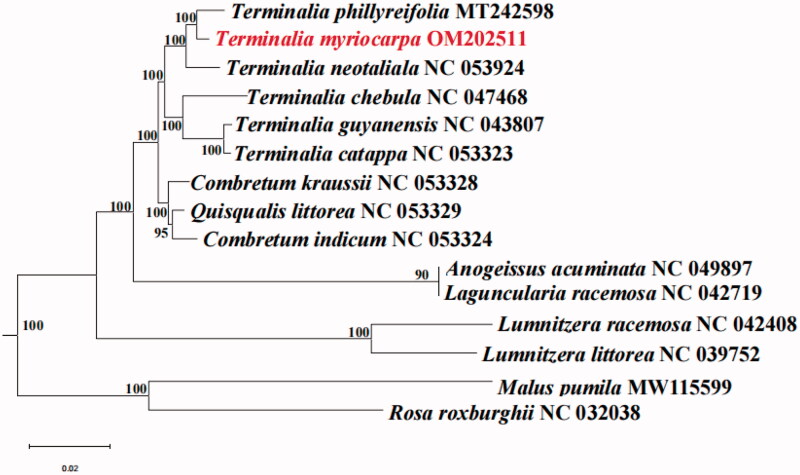
Maximum-likelihood phylogenetic tree was reconstructed by RAxML based on complete plastid genome sequences from 11 Combretaceae species (*T. myriocapra* in this study and 10 previously reported species). Numbers on branches are bootstrap support values.

## Authors’ contributions

L. L. D. conceived the study; X. Y. collected the molecular materials and drafted the manuscript; Z. N. Z. and H. L. P. analyzed the experimental data; L. L. D. revised the manuscript. All authors provided comments and final approval.

## Data Availability

The genome sequence data that support the findings of this study are openly available in GenBank of NCBI at https://www.ncbi.nlm.nih.gov under the accession no.OM202511. The associated BioProject, SRA, and Bio-Sample numbers are PRJNA797285, SRR17621105, and SAMN24979394 respectively.

## References

[CIT0001] Beier S, Thiel T, Münch T, Scholz U, Mascher M. 2017. MISA-web: a web server for microsatellite prediction. Bioinformatics. 33(16):834–2585. 28398459 10.1093/bioinformatics/btx198 PMC5870701

[CIT0002] Doyle JJ, Doyle JL. 1987. A rapid DNA isolation procedure for small amounts of fresh leaf tissue. Phytochem Bull. 19(1):11–15.

[CIT0003] Jin J-J, Yu W-B, Yang J-B, Song Y, dePamphilis CW, Yi T-S, Li D-Z. 2020. GetOrganelle: a fast and versatile toolkit for accurate de novo assembly of organelle genomes. Genome Biol. 21(1):1–31. 10.1186/s13059-020-02154-5 PMC7488116 32912315

[CIT0004] Kearse M, Moir R, Wilson A, Stones-Havas S, Cheung M, Sturrock S, Buxton S, Cooper A, Markowitz S, Duran C, et al. 2012. Geneious Basic: an integrated and extendable desktop software platform for the organization and analysis of sequence data. Bioinformatics. 28(12):1647–1649. 22543367 10.1093/bioinformatics/bts199 PMC3371832

[CIT0005] Katoh K, Standley DM. 2013. MAFFT multiple sequence alignment software version 7: improvements in performance and usability. Mol Biol Evol. 30(4):772–780. 23329690 10.1093/molbev/mst010 PMC3603318

[CIT0006] Li YY. 2004. National key protected wild plants in Yunnan Province. Kunming (China): Yunnan Science and Technology Press; p. 155–156.

[CIT0007] Stamatakis A. 2014. RAxML version 8: a tool for phylogenetic analysis and post-analysis of large phylogenies. Bioinformatics. 30(9):1312–1313. 24451623 10.1093/bioinformatics/btu033 PMC3998144

[CIT0008] Lu J, Zhou CF, Xu BY, Chen HW, Chen W. 2010. Preliminary study on breeding of *Terminalia myriocarpa*. J West China Forest Sci. 39(01):81–85.

[CIT0009] Wu ZY. 1984. *Terminalia myriocapra*. In: Flora of China. Beijing (China): Science Press; p. 7.

[CIT0010] Yu GX, Han L, Dong SF, Yang LQ. 2017. Effects of exterior hormones and scion on survival rate of *Terminalia myriocarpa* cutting seedlings. J West China Forestry Sci. 46(01):44–48.

[CIT0011] Yu X, Deng LL, Yang ZY, Zhao JM. 2017. A survey of resources of *Terminalia myriocarpa* in Ruili County and its landscape application. J Hubei Univ Nation. 35(01):97–100.

[CIT0012] Yu X, Xin PY, Yin YM, Yang ZY, Deng LL. 2019. The karyotype analysis of *Terminalia myriocarpa* and *Terminalia neotaliala*. J Fujian Forest Sci Technol. 46(03):46–50.

[CIT0013] Yu X, Zhang B, Li WF, Deng LL. 2020. Study on population characteristics and community characteristics of endangered species *Terminalia myriocapra*. J Hubei Minzu Univ. 38(03):257–263.

[CIT0014] Yu X, Zhao ZN, Ping HL, Deng LL. 2022. Effects of exogenous hormones on cutting rooting of endangered species *Terminalia myriocapra*. J Hubei Minzu Univ. 40(1):1–9.

